# 2-Fluoro-*N*′-(2-hy­droxy­benzyl­idene)benzohydrazide

**DOI:** 10.1107/S1600536810050841

**Published:** 2010-12-11

**Authors:** Cheng-Bi Xu, Zong-Gui Wang, Yi Nan, Ling Yuan, Rong Wang, Shu-Xiang Zhang

**Affiliations:** a The Second Hospital of Jilin University, Changchun Jilin 130041, People’s Republic of China; b Traditional Chinese Medicine College of Ningxia Medical University, Yinchuan Ningxia 750004, People’s Republic of China; c Pharmacy College of Ningxia Medical University, Yinchuan Ningxia 750004, People’s Republic of China; d Minority Traditional Medical Center of Minzu University of China, Beijing 100081, People’s Republic of China; e Affiliated Hospital of Ningxia Medical University, Yinchuan Ningxia 750004, People’s Republic of China

## Abstract

In the title compound, C_14_H_11_FN_2_O_2_, an intra­molecular O—H⋯N hydrogen bond influences the mol­ecular conformation; the two benzene rings form a dihedral angle of 18.4 (3)°. The F atom is disordered over two positions in a 0.717 (5):0.283 (5) ratio. In the crystal, inter­molecular N—H⋯O hydrogen bonds link the mol­ecules into chains extending along the *c* axis.

## Related literature

For the reference bond lengths, see: Allen *et al.* (1987 ▶). For structural studies of hydrazone compounds, see: Han & Zhao (2010 ▶); Zhou & Yang (2010 ▶); Huang & Wu (2010 ▶); Shalash *et al.* (2010 ▶). For a related structure, see: Xu *et al.* (2011 ▶).
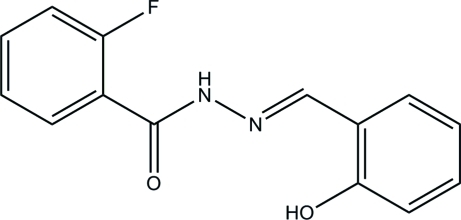


## Experimental

### 

#### Crystal data

C_14_H_11_FN_2_O_2_
*M* *_r_* = 258.25
Monoclinic, 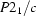

*a* = 10.661 (3) Å
*b* = 13.515 (3) Å
*c* = 8.998 (3) Å
β = 98.150 (3)°
*V* = 1283.4 (6) Å^3^
*Z* = 4
Mo *K*α radiation
μ = 0.10 mm^−1^
*T* = 298 K
0.20 × 0.20 × 0.17 mm

#### Data collection

Bruker SMART CCD area-detector diffractometer
Absorption correction: multi-scan (*SADABS*; Sheldrick, 1996 ▶) *T*_min_ = 0.980, *T*_max_ = 0.983
6829 measured reflections
2675 independent reflections
1093 reflections with *I* > 2σ(*I*)
*R*_int_ = 0.066

#### Refinement

*R*[*F* ^2^ > 2σ(*F* ^2^)] = 0.064
*wR*(*F* ^2^) = 0.198
*S* = 0.97
2675 reflections
180 parameters
1 restraint
H-atom parameters constrained
Δρ_max_ = 0.37 e Å^−3^
Δρ_min_ = −0.18 e Å^−3^

### 

Data collection: *SMART* (Bruker, 1998 ▶); cell refinement: *SAINT* (Bruker, 1998 ▶); data reduction: *SAINT*; program(s) used to solve structure: *SHELXS97* (Sheldrick, 2008 ▶); program(s) used to refine structure: *SHELXL97* (Sheldrick, 2008 ▶); molecular graphics: *SHELXTL* (Sheldrick, 2008 ▶); software used to prepare material for publication: *SHELXTL*.

## Supplementary Material

Crystal structure: contains datablocks global, I. DOI: 10.1107/S1600536810050841/cv5010sup1.cif

Structure factors: contains datablocks I. DOI: 10.1107/S1600536810050841/cv5010Isup2.hkl

Additional supplementary materials:  crystallographic information; 3D view; checkCIF report

## Figures and Tables

**Table 1:** Hydrogen-bond geometry (Å, °)

*D*—H⋯*A*	*D*—H	H⋯*A*	*D*⋯*A*	*D*—H⋯*A*
O1—H1⋯N1	0.82	1.92	2.637 (3)	146
N2—H2⋯O2^i^	0.86	2.02	2.841 (3)	158

Symmetry code: (i) 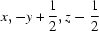.

**Fig. 1.**
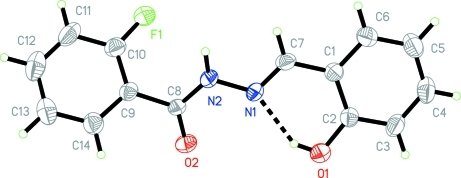
The molecular structure of the title compound, with displacement ellipsoids drawn at the 30% probability level. The disordered fluorine atom F1 is shown in the major position. Dashed line denotes hydrogen bond.

**Fig. 2.**
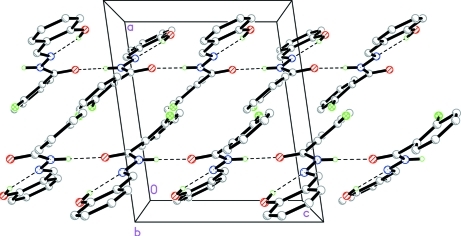
A portion of the crystal packing showing hydrogen-bonded (dashed lines) one-dimensional chain of the molecules. H atoms not involved in hydrogen-bonding omitted for clarity.

## supplementary crystallographic information

### Comment

As a contribution to a structural study of hydrazone compounds (Han & Zhao, 2010; Zhou & Yang, 2010; Huang & Wu, 2010; Shalash *et al.*, 2010), we present here the crystal structure of the title compound.

There is an intramolecular O—H···N hydrogen bond (Table 1) in the molecule of the title compound (Fig. 1). The molecule exists in a *trans* configuration with respect to the methylidene unit. The molecule is twisted, with the dihedral angle between the two benzene rings of 18.4 (3)°. The torsion angle C7—N1—N2—C8 is 8.1 (3)°. The bond lengths are within normal ranges (Allen *et al.*, 1987) and are comparable with those observed in the similar compounds (Xu *et al.*, 2011).

In the crystal structure, molecules are linked through intermolecular N—H···O hydrogen bonds (Table 1), to form chains down the *c* axis (Fig. 2).

### Experimental

Salicylaldehyde (0.1 mmol, 12.2 mg) and 2-fluorobenzohydrazide (0.1 mmol, 15.4 mg) were mixed in ethanol (20 ml). The mixture was stirred at room temperature to give a clear colorless solution. Colorless well shaped crystals of the title compound were formed by gradual evaporation of the solvent over a period of three days at room temperature.

### Refinement

All H atoms were placed in geometrically idealized positions, with C—H = 0.93 Å, O—H = 0.82 Å, N—H = 0.86 Å, and refined as riding, with U_iso_(H) = 1.2U_eq_(C,N) and 1.5U_eq_(O). Atom F1 has been refined as disordered between two positions in a ratio  0.717 (5):0.283 (5) being attached either to C10 or C14, respectively.

### Crystal data

**Table:**

C_14_H_11_FN_2_O_2_	*F*(000) = 536
*M**_r_* = 258.25	*D*_x_ = 1.337 Mg m^−^^3^
Monoclinic, *P*2_1_/*c*	Mo *K*α radiation, λ = 0.71073 Å
Hall symbol: -P 2ybc	Cell parameters from 620 reflections
*a* = 10.661 (3) Å	θ = 2.4–24.3°
*b* = 13.515 (3) Å	µ = 0.10 mm^−^^1^
*c* = 8.998 (3) Å	*T* = 298 K
β = 98.150 (3)°	Block, colourless
*V* = 1283.4 (6) Å^3^	0.20 × 0.20 × 0.17 mm
*Z* = 4	

### Data collection

**Table:**

Bruker SMART CCD area-detector diffractometer	2675 independent reflections
Radiation source: fine-focus sealed tube	1093 reflections with *I* > 2σ(*I*)
graphite	*R*_int_ = 0.066
ω scans	θ_max_ = 27.0°, θ_min_ = 2.5°
Absorption correction: multi-scan (*SADABS*; Sheldrick, 1996)	*h* = −13→11
*T*_min_ = 0.980, *T*_max_ = 0.983	*k* = −17→15
6829 measured reflections	*l* = −11→10

### Refinement

**Table:**

Refinement on *F*^2^	Primary atom site location: structure-invariant direct methods
Least-squares matrix: full	Secondary atom site location: difference Fourier map
*R*[*F*^2^ > 2σ(*F*^2^)] = 0.064	Hydrogen site location: inferred from neighbouring sites
*wR*(*F*^2^) = 0.198	H-atom parameters constrained
*S* = 0.97	*w* = 1/[σ^2^(*F*_o_^2^) + (0.0776*P*)^2^] where *P* = (*F*_o_^2^ + 2*F*_c_^2^)/3
2675 reflections	(Δ/σ)_max_ < 0.001
180 parameters	Δρ_max_ = 0.37 e Å^−^^3^
1 restraint	Δρ_min_ = −0.18 e Å^−^^3^

### Special details

**Table:**

Geometry. All esds (except the esd in the dihedral angle between two l.s. planes) are estimated using the full covariance matrix. The cell esds are taken into account individually in the estimation of esds in distances, angles and torsion angles; correlations between esds in cell parameters are only used when they are defined by crystal symmetry. An approximate (isotropic) treatment of cell esds is used for estimating esds involving l.s. planes.
Refinement. Refinement of F^2^ against ALL reflections. The weighted R-factor wR and goodness of fit S are based on F^2^, conventional R-factors R are based on F, with F set to zero for negative F^2^. The threshold expression of F^2^ > 2sigma(F^2^) is used only for calculating R-factors(gt) etc. and is not relevant to the choice of reflections for refinement. R-factors based on F^2^ are statistically about twice as large as those based on F, and R- factors based on ALL data will be even larger.

### Fractional atomic coordinates and isotropic or equivalent isotropic displacement parameters (Å^2^)

**Table:**

	*x*	*y*	*z*	*U*_iso_*/*U*_eq_	Occ. (<1)
N1	0.7863 (3)	0.15833 (19)	1.0481 (3)	0.0527 (8)	
N2	0.7326 (3)	0.23495 (19)	0.9579 (3)	0.0576 (8)	
H2	0.7179	0.2280	0.8620	0.069*	
O1	0.9198 (3)	0.08661 (17)	1.2936 (2)	0.0711 (8)	
H1	0.8803	0.1289	1.2408	0.107*	
O2	0.7248 (2)	0.33336 (17)	1.1581 (2)	0.0712 (8)	
C1	0.8526 (3)	−0.0093 (2)	1.0701 (3)	0.0506 (9)	
C2	0.9105 (3)	−0.0011 (2)	1.2207 (4)	0.0527 (9)	
C3	0.9606 (3)	−0.0842 (3)	1.2979 (4)	0.0717 (11)	
H3	1.0004	−0.0782	1.3964	0.086*	
C4	0.9518 (4)	−0.1747 (3)	1.2298 (5)	0.0804 (12)	
H4	0.9841	−0.2300	1.2835	0.096*	
C5	0.8961 (4)	−0.1856 (3)	1.0833 (5)	0.0809 (12)	
H5	0.8911	−0.2477	1.0382	0.097*	
C6	0.8476 (3)	−0.1033 (3)	1.0036 (4)	0.0692 (11)	
H6	0.8111	−0.1104	0.9040	0.083*	
C7	0.7984 (3)	0.0748 (2)	0.9849 (3)	0.0554 (9)	
H7	0.7720	0.0681	0.8824	0.067*	
C8	0.7038 (3)	0.3200 (2)	1.0222 (3)	0.0499 (9)	
C9	0.6465 (3)	0.3994 (2)	0.9199 (3)	0.0511 (9)	
C11	0.5089 (4)	0.4606 (4)	0.7014 (4)	0.0849 (13)	
H11	0.4522	0.4476	0.6152	0.102*	
C12	0.5415 (4)	0.5549 (4)	0.7403 (5)	0.0922 (14)	
H12	0.5064	0.6068	0.6805	0.111*	
C13	0.6252 (3)	0.5749 (2)	0.8661 (3)	0.0853 (13)	
H13	0.6484	0.6397	0.8912	0.102*	
C10	0.5613 (3)	0.3835 (2)	0.7917 (3)	0.0668 (10)	
H10	0.5381	0.3188	0.7649	0.080*	0.283 (5)
F1'	0.7513 (8)	0.5218 (5)	1.0737 (9)	0.100 (4)	0.283 (5)
C14	0.6747 (4)	0.4973 (3)	0.9556 (4)	0.0670 (10)	
H14A	0.7291	0.5114	1.0432	0.080*	0.717 (5)
F1	0.5221 (3)	0.2946 (2)	0.7509 (3)	0.0909 (14)	0.717 (5)

### Atomic displacement parameters (Å^2^)

**Table:**

	*U*^11^	*U*^22^	*U*^33^	*U*^12^	*U*^13^	*U*^23^
N1	0.0652 (19)	0.0501 (16)	0.0403 (15)	0.0014 (14)	−0.0004 (13)	0.0089 (14)
N2	0.081 (2)	0.0543 (17)	0.0348 (14)	0.0059 (15)	−0.0007 (14)	0.0064 (13)
O1	0.092 (2)	0.0656 (16)	0.0514 (14)	0.0041 (14)	−0.0058 (13)	−0.0005 (12)
O2	0.098 (2)	0.0753 (17)	0.0376 (13)	0.0060 (13)	0.0017 (12)	−0.0036 (12)
C1	0.056 (2)	0.051 (2)	0.0455 (19)	−0.0016 (16)	0.0106 (16)	0.0028 (16)
C2	0.054 (2)	0.055 (2)	0.050 (2)	0.0039 (17)	0.0106 (17)	0.0028 (17)
C3	0.074 (3)	0.078 (3)	0.060 (2)	0.022 (2)	0.0000 (19)	0.012 (2)
C4	0.097 (3)	0.066 (3)	0.081 (3)	0.027 (2)	0.023 (3)	0.020 (2)
C5	0.105 (3)	0.055 (2)	0.085 (3)	0.010 (2)	0.022 (3)	−0.003 (2)
C6	0.086 (3)	0.062 (2)	0.059 (2)	0.001 (2)	0.006 (2)	−0.0040 (19)
C7	0.067 (2)	0.059 (2)	0.0388 (18)	−0.0080 (18)	−0.0002 (16)	0.0029 (17)
C8	0.063 (2)	0.050 (2)	0.0355 (18)	−0.0019 (16)	0.0023 (16)	0.0002 (15)
C9	0.059 (2)	0.058 (2)	0.0383 (17)	0.0054 (17)	0.0115 (16)	0.0036 (15)
C11	0.089 (3)	0.104 (4)	0.059 (2)	0.041 (3)	−0.001 (2)	0.011 (2)
C12	0.105 (4)	0.090 (4)	0.087 (3)	0.040 (3)	0.031 (3)	0.030 (3)
C13	0.101 (4)	0.059 (3)	0.100 (3)	0.009 (2)	0.025 (3)	0.008 (2)
C10	0.075 (3)	0.074 (3)	0.050 (2)	0.005 (2)	0.005 (2)	−0.0020 (19)
F1'	0.115 (7)	0.075 (6)	0.097 (7)	−0.002 (5)	−0.025 (5)	−0.006 (5)
C14	0.076 (3)	0.062 (2)	0.064 (2)	0.006 (2)	0.013 (2)	0.001 (2)
F1	0.096 (3)	0.074 (2)	0.088 (2)	0.0046 (17)	−0.0376 (18)	−0.0071 (17)

### Geometric parameters (Å, °)

**Table:**

N1—C7	1.278 (4)	C6—H6	0.9300
N1—N2	1.389 (3)	C7—H7	0.9300
N2—C8	1.342 (4)	C8—C9	1.487 (4)
N2—H2	0.8600	C9—C10	1.381 (4)
O1—C2	1.351 (4)	C9—C14	1.384 (5)
O1—H1	0.8200	C11—C12	1.354 (5)
O2—C8	1.225 (3)	C11—C10	1.389 (4)
C1—C6	1.402 (4)	C11—H11	0.9300
C1—C2	1.412 (4)	C12—C13	1.365 (5)
C1—C7	1.445 (4)	C12—H12	0.9300
C2—C3	1.387 (4)	C13—C14	1.380 (4)
C3—C4	1.366 (5)	C13—H13	0.9300
C3—H3	0.9300	C10—F1	1.307 (4)
C4—C5	1.375 (5)	C10—H10	0.9300
C4—H4	0.9300	F1'—C14	1.288 (7)
C5—C6	1.384 (5)	C14—H14A	0.9300
C5—H5	0.9300		
			
C7—N1—N2	117.2 (2)	O2—C8—N2	122.4 (3)
C8—N2—N1	119.2 (2)	O2—C8—C9	120.8 (3)
C8—N2—H2	120.4	N2—C8—C9	116.8 (3)
N1—N2—H2	120.4	C10—C9—C14	116.0 (3)
C2—O1—H1	109.5	C10—C9—C8	124.6 (3)
C6—C1—C2	117.8 (3)	C14—C9—C8	119.3 (3)
C6—C1—C7	119.9 (3)	C12—C11—C10	119.2 (4)
C2—C1—C7	122.4 (3)	C12—C11—H11	120.4
O1—C2—C3	118.1 (3)	C10—C11—H11	120.4
O1—C2—C1	121.8 (3)	C11—C12—C13	120.9 (4)
C3—C2—C1	120.1 (3)	C11—C12—H12	119.5
C4—C3—C2	120.2 (3)	C13—C12—H12	119.5
C4—C3—H3	119.9	C12—C13—C14	119.0 (3)
C2—C3—H3	119.9	C12—C13—H13	120.5
C3—C4—C5	121.3 (4)	C14—C13—H13	120.5
C3—C4—H4	119.3	F1—C10—C9	121.6 (3)
C5—C4—H4	119.3	F1—C10—C11	116.1 (3)
C4—C5—C6	119.3 (4)	C9—C10—C11	122.3 (3)
C4—C5—H5	120.4	C9—C10—H10	118.9
C6—C5—H5	120.4	C11—C10—H10	118.9
C5—C6—C1	121.3 (3)	F1'—C14—C13	115.6 (5)
C5—C6—H6	119.4	F1'—C14—C9	121.9 (5)
C1—C6—H6	119.4	C13—C14—C9	122.5 (4)
N1—C7—C1	121.1 (3)	C13—C14—H14A	118.7
N1—C7—H7	119.5	C9—C14—H14A	118.7
C1—C7—H7	119.5		

### Hydrogen-bond geometry (Å, °)

**Table:**

*D*—H···*A*	*D*—H	H···*A*	*D*···*A*	*D*—H···*A*
O1—H1···N1	0.82	1.92	2.637 (3)	146.
N2—H2···O2^i^	0.86	2.02	2.841 (3)	158.4

Symmetry codes: (i) *x*, −*y*+1/2, *z*−1/2.
